# Implementation of evidence-based care bundles in the ICU

**DOI:** 10.1186/cc11131

**Published:** 2012-03-20

**Authors:** G Juknevicius, E Balakumar, A Gratrix

**Affiliations:** 1 Hull Royal Infirmary, Hull, UK

## Introduction

Implementation of an evidence-based care bundle in critically ill patients has been shown to improve outcome. Use of care bundles to reduce ventilator-associated pneumonia and other ICU complications has been increasing in critical care practice.

## Methods

We conducted a prospective audit on implementation of a care bundle after audit approval. We collected data for 101 patient days from all patients admitted to Hull Royal Infirmary ICU during the month of November 2011. We collected information regarding stress ulcer prophylaxis, deep vein thrombosis (DVT) prophylaxis, ventilator care bundle, blood glucose control, daily assessment of need for a central line, sedation score assessment and delirium score assessment at least twice a day.

## Results

All patients received stress ulcer prophylaxis. At least 95% of patients received DVT prophylaxis, adequate blood glucose control and appropriate sedation need assessment. There was further scope for improvement in areas of sedation hold practice and assessing daily need for a central line. Poor clinical practice was identified in delirium score assessment and head elevation to reduce VAP. See Table 1.

**Table 1:** 

Intervention in eligible patients	**Adherence**, *n ***(%)**
Stress ulcer prophylaxis	101/101 (100)
DVT/PE prophylaxis	94/97 (97)
Head elevation 30% in ventilated patients	62/75 (83)
Daily sedation hold	28/32 (88)
Blood glucose control	96/101 (95)
Need for central line assessed	73/85 (86)
Sedation score assessment	98/101 (97)
CAM-ICU score at least twice a day	29/101 (28)

## Conclusion

It is very challenging to implement care bundles despite evidence showing that they improve outcome. A recent study suggests that doing a daily quality rounds checklist (QRC) will improve long-term compliance, thereby reducing potential complications for intensive care patients [1]. We have implemented QRC in our practice and will be re-auditing in 6 months to ensure continued adherence.

## References

[B1] DuBose Measurable outcomes of quality improvement in the trauma intensive care unit: the impact of a daily quality rounding checklist J Trauma 2008 64 22 29 10.1097/TA.0b013e318161b0c8 18188094

